# Evaluation of Antimicrobial Resistancein Clinical Isolates of *Enterococcus* spp. Obtained from Hospital Patients in Latvia

**DOI:** 10.3390/medicina60060850

**Published:** 2024-05-23

**Authors:** Linda Labecka, Juris Ķibilds, Aivars Cīrulis, Evelīna Diāna Čeirāne, Indra Zeltiņa, Aigars Reinis, Barba Vilima, Dace Rudzīte, Renārs Erts, Inga Mauliņa, Dace Bandere, Angelika Krūmiņa

**Affiliations:** 1 Institute of Food Safety, Animal Health and Environment “BIOR”, Lejupes Street 3, LV-1076 Riga, Latviaaivars.cirulis@bior.lv (A.C.); krumina.angelika@inbox.lv (A.K.); 2 Faculty of Biology, University of Latvia, Jelgavas Street 1, LV-1004 Riga, Latvia; 3 Faculty of Medicine, Riga Stradins University, Dzirciema Street 16, LV-1007 Riga, Latvia; 4 Department of Infectology, Riga Stradiņš University, Dzirciema Street 16, LV-1007 Riga, Latvia; indra.zeltina@rsu.lv; 5 Riga East Clinical University Hospital, Hipokrata Street 2, LV-1038 Riga, Latvia; dace.rudzite@aslimnica.lv (D.R.); ingamaulina@inbox.lv (I.M.); 6 Department of Biology and Microbiology, Riga Stradiņš University, Dzirciema Street 16, LV-1007 Riga, Latvia; aigars.reinis@rsu.lv; 7 Pauls Stradiņš Clinical University Hospital, Pilsoņu Street 13, LV-1002 Riga, Latvia; 8 Vidzeme Hospital, Jumaras Street 195, LV-4201 Valmiera, Latvia; barba.vilima@vidzemesslimnica.lv; 9 Faculty of Medicine, University of Latvia, Raiņa bulvāris 19, LV-1586 Riga, Latvia; renars.erts@gmail.com; 10 Department of Pharmaceutical Chemistry, Riga Stradiņš University, LV-1007 Riga, Latvia; dace.bandere@rsu.lv; 11 Baltic Biomaterials Centre of Excellence, Headquarters at Riga Technical University, LV-1658 Riga, Latvia

**Keywords:** enterococci, antimicrobial resistance, VRE, *van*B, LRE, resistance genes

## Abstract

*Background and Objective*: Enterococci are typically found in a healthy human gastrointestinal tract but can cause severe infections in immunocompromised patients. Such infections are treated with antibiotics. This study addresses the rising concern of antimicrobial resistance (AMR) in Enterococci, focusing on the prevalence of vancomycin-resistant enterococcus (VRE) strains. *Materials and Methods*: The pilot study involved 140 Enterococci isolates collected between 2021 and 2022 from two multidisciplinary hospitals (with and without local therapeutic drug monitoring protocol of vancomycin) in Latvia. Microbiological assays and whole genome sequencing were used. AMR gene prevalence with resistance profiles were determined and the genetic relationship and outbreak evaluation were made by applying core genome multi-locus sequence typing (cgMLST). *Results*: The acquired genes and mutations were responsible for resistance against 10 antimicrobial classes, including 25.0% of isolates expressing resistance to vancomycin, predominantly of the *van*B type. Genetic diversity among *E. faecalis* and *E. faecium* isolates was observed and seven potential outbreak clusters were identified, three of them containing sequence types ST6, ST78 and ST80. The prevalence of vancomycin resistance was highest in the hospital without a therapeutic drug-monitoring protocol and in *E. faecium*. Notably, a case of linezolid resistance due to a mutation was documented. *Conclusions*: The study illustrates the concerning prevalence of multidrug-resistant Enterococci in Latvian hospitals, showcasing the rather widespread occurrence of vancomycin-resistant strains. This highlights the urgency of implementing efficient infection control mechanisms and the need for continuous VRE surveillance in Latvia to define the scope and pattern of the problem, influencing clinical decision making and planning further preventative measures.

## 1. Introduction

*Enterococcus* is a large genus of Gram-positive, facultative anaerobic cocci, which can be isolated from animals, birds, plants and various environmental sources like soil and water. In humans and animals, enterococci are commonly found as commensal microorganisms in the gastrointestinal tract. Enterococci are also known to be capable of spreading rapidly and adapting quickly to unfavourable and changing environmental conditions. Mechanisms regarding adaptation, whether inherent or gained, can manifest at either an individual or population level. This rapid rate of adaptation is possible mostly due to acquired resistance through mutations or the acquisition of foreign, valuable genetic material [1]. The adaptation of enterococci to thrive in challenging environmental conditions has played a major role in their increased prevalence after the introduction of antimicrobial therapy. The ability of bacteria to withstand antimicrobials can be influenced by both internal and external factors, with adaptation playing an important role [2]. Due to this adaptation, enterococci have emerged as significant nosocomial opportunistic pathogens, responsible for causing bacteraemia, endocarditis, urinary tract infections, wound infections, or intra-abdominal infections, and are one of the most commonly isolated pathogens in hospital settings [1,3]. The adaptability of Enterococci together with their potential pathogenicity creates a considerable challenge for the controlling of resistant bacteria from spreading within a hospital environment [4,5].

Two of the most common Enterococcal species causing bacterial infections are *E. faecalis* and *E. faecium*. Clinically detected *E. faecalis* and *E. faecium* species usually have some innate resistance to antibiotics. Enterococci can have innate resistance against aminoglycosides, macrolides, β-lactams and lincosamides (*E. faecalis*), meaning they are commonly found to be multidrug resistant [1,6,7].

In cases of severe infections where first- and second-line antibiotics are not efficient, reserve antibiotics are administered to treat an infection. One such example is a vancomycin–glycopeptide class drug, discovered in the middle of the 20th century, but not used widely until the 1980s [8]. Soon after the introduction of vancomycin, microbial resistance was detected, and within the last decade, it has been a serious matter for concern, due to its increasing resistance ever since, especially in the two most common species of *Enterococcus* [9]. The genomic flexibility of vancomycin-resistant enterococci has resulted in the evolution of a specific clade of *E. faecium* within hospitals, exhibiting resistance to multiple antibiotics [3].

As vancomycin-resistant *Enterococcus* is a global health concern, it is important to keep monitoring the resistance rate of such strains and provide valuable information to competent authorities for the surveillance of public health. In all cases of antibiotic-resistant bacteria, it is crucial to determine a pattern of antibacterial resistance, emerging trends, prevalence and scope of the problem, to implement controlling mechanisms accordingly, adjust antibacterial therapy and design further preventative measures to contain the spread of the pathogenic bacteria; therefore, by decreasing both, the risk of infections and further spread of antibiotic resistance is limited.

The aim of our pilot study was to detect all species of Enterococci obtained from the microbiological assays in two multidisciplinary hospitals—“A”, a hospital without a local therapeutic drug monitoring (TDM) protocol of vancomycin and “B”, a hospital with a local vancomycin TDM protocol in Latvia—to evaluate the genetic background of each *Enterococcus* isolates by applying whole genome sequencing, to further analyse the data of all antimicrobial genes, predictable phenotypic antibacterial resistance and the prevalence of antimicrobial resistance (AMR) genes in both hospitals. A core genome multi-locus sequence typing (cgMLST) was applied to evaluate possible outbreaks and determine sequence types of the two most common *Enterococcus* bacteria found in hospital settings—*E. faecalis* and *E. faecium*.

The two hospitals were selected based on the fact that both have multidisciplinary profiles and an already established relation (the exchange rate of patients between both hospitals according to the general plan of the structure of the healthcare system in the Republic of Latvia).

## 2. Materials and Methods

### 2.1. Samples and Sampling

Our pilot study investigated the genetic variations using a random sampling approach. We selected individuals from two hospitals—A and B—without considering their population sizes or number of hospital beds. This method ensured everyone had an equal chance of being chosen, which is essential for random sampling.

One hundred and forty-four (144) samples from patients in two hospitals in Latvia (for the purpose of anonymity referred to as “A” and “B”), were collected and analysed using a random sampling approach from 2021 to 2022. Both are tertiary-level hospitals. Four of the samples were later eliminated because of inferior quality for genetic testing or contamination. Since the year 2018, hospital “B” additionally uses internally approved vancomycin TDM protocols in their clinical practice. Therapeutic drug monitoring is the process of measuring the concentration of specific medicines to maintain a constant concentration in plasma, and then adjusting the therapy—both doses and dosage intervals—based on the results of the plasma concentration. As a part of this study, Enterococci were more commonly isolated from blood, faeces, urine or wounds. The study was approved by the Ethics Committee of Riga Stradins University, 16 Dzirciema Str., LV-1007, Riga, Latvia, approval no. 6-1/09/11, 10 September 2020.

### 2.2. Microbiological Testing of Samples

In hospital “A”, laboratory-isolated microorganisms were cultivated on 5% blood agar plates (Columbia Blood Agar Base, HiMedia, Laboratories, Maharashtra, India), supplemented with sheep blood, and defibrinated (TCS Biosciences Ltd., Buckingham, UK). Microorganism identification was performed using Matrix Assisted Laser Desorption Ionization Time-of-Flight (MALDI-TOF) Vitek MS (bioMérieux, Craponne, France) and an antimicrobial susceptibility test to determine the antimicrobial susceptibility was performed using disk diffusion method (Liofilchem, Roseto degli Abruzzi, Italy) and interpreted according to the European Committee on Antimicrobial Susceptibility Testing (EUCAST) [10] criteria at the time of testing. Microorganism identification and an antimicrobial susceptibility test to determine the antimicrobial susceptibility were performed using Vitek-2 system (bioMérieux, France) by applying GP card for identification and AST 643 susceptibility cards for phenotypic susceptibility. Vitek-2 analyser automated susceptibility testing was performed with systems validated for use with European breakpoints, according to the European Committee on Antimicrobial Susceptibility Testing (EUCAST) [10] criteria at the time of testing.

One sample was retested for antibiotic susceptibility with broth microdilution method to confirm the results of whole genome sequencing.

### 2.3. Extraction of DNA and Whole-Genome Sequencing (WGS)

Before sequencing, microorganisms were again identified using MALDI-TOF (Bruker, Ettlingen, Germany) mass spectrometer. DNA of isolates was extracted using NucleoSpin Tissue kit (Macherey-Nagel, Düren, Germany) adding additional lysozyme enzyme for breaking down the cell wall, and then following the standard protocol nr.5. The purity of the samples was assessed using a NanoDrop spectrophotometer and the quantity was measured using a Qubit fluorometer (both ThermoFisher Scientific, Landsmeer, The Netherlands). Libraries for WGS were prepared with Illumina DNA prep (Illumina, San Diego, CA, USA) following the provided instructions. Sample quality before pooling was assessed with the gel capillary electrophoresis (QIAxcel Advanced Instrument, QIAGEN, Venlo, Limburg, The Netherlands) using a high-resolution cartridge and a 50–1500 bp size marker. Whole genome sequencing was performed with the next-generation Illumina MiSeq sequencer (Illumina, Sand Diega, CA, USA).

### 2.4. Genome Assembly, Detection of Resistance Genes and Core Genome Sequence Typing

Raw data files were uploaded, genome assembled and data analysed using publicly available sources in Galaxy platform [11] and CGE website (Center for Genomic Epidemiology) [12] using most recent databases at the time. The quality of the raw files was assessed using FastQC (Galaxy Version 0.72) and trimmed using Trimmomatic tool (Galaxy Version 0.38.1) with the following settings: LEADING:17 TRAILING:0 SLIDINGWINDOW:4:20 MINLEN:30, and then assembled using SPAdes or Shovill with estimated genome size set to 2.5–3.5 Mb. Afterwards, QUAST was used to assess the quality of the assemblies. During this stage, four samples were eliminated due to contamination or poor quality. Within Galaxy platform, genome analysis was conducted using “staramr” program (version 0.9.1) and the following databases: ResFinder (24 May 2022, v072621), PointFinder (1 February 2021, v072621.1) and PlasmidFinder (https://cge.food.dtu.dk/services/PlasmidFinder/, 29 November 2021). Gene location on mobile genetic elements and information regarding linezolid resistance was assessed through CGE website using Mobile Genetic Elements (MGE) (software version: v1.0.3 (9 October 2020), database version: v1.0.2 (9 June 2020) and LRE-Finder (version 1.0) accessed in March 2023. Core genome multilocus sequence typing (cgMLST) with Ridom SeqSphere+ programme (Ridom, Muenster, Germany) was also performed to evaluate transmission paths and monitor occurrence of the outbreaks [13]. For easier analysis and outbreak investigation of data created with cgMLST, it was displayed and analysed with freely available minimum-spanning tree visualization program “GrapeTree” using the MSTreeV2 algorithm (https://achtman-lab.github.io/GrapeTree/MSTree_holder.html accessed on 21 January 2024) [14].

All information regarding sample names, resistance genes, sequence types, mutations, isolation sources, hospital and year of isolation can be found in the table under Supplementary Material section.

### 2.5. Statistical Analysis

To see if vancomycin resistance significantly differs between the hospitals or species, G-test from package DescTools (version 0.99.54) in R (version 4.3.2) R Core Team (2023) was used.

## 3. Results

### 3.1. Antimicrobial Resistance Genes in Enterococcus Isolates

Four of the isolated samples were eliminated due to inferior quality for genetic testing or contamination. Within the samples collected from both hospitals, five different species of Enterococci were detected: *E. faecalis* (n = 82), *E. faecium* (n = 55), *E. gallinarum* (n = 1), *E. avium* (n = 1) and *E. durans* (n = 1). 79.3% (65/82) of *E. faecalis* isolates, 100% (55/55) of *E. faecium* isolates and the only isolate of *E. avium* were multiresistant. A total of 20 antimicrobial genes were identified according to the ResFinder database on WGS data (Table 1). These different antibiotic resistance genes determine resistance against seven different classes of antibacterial agents: aminoglycosides, macrolides, lincosamides, glycopeptides, tetracyclines, trimethoprimes and chloramphenicols. In addition, the stress tolerance gene *clp*L was frequently observed.

Within *E. faecalis* isolates, the *clp*L gene was found in seventeen samples. Fifteen of these genes were located in insertion sequences (IS). Thirteen were found in ISLla3, one in ISLgar3 and another in ISEfm2. The *cIp*L gene was also detected in seven samples of *E. faecium*, and in four cases it was located on one sequence read (contig) with plasmid replicon *rep1*, leading to the belief that it is in the plasmid DNA [15].

Mutations in *gyr*A and *par*C genes with a substitution of one nucleotide in the 83rd and 80th or 87th and 80th (one case) positions were also noticed. This type of mutation induces fluoroquinolone antibiotic resistance. Many cases of mutations in penicillin-binding proteins-5 (PBP-5) in *E. faecium* were also noticed, which leads to penicillin (ampicillin) resistance. One case of mutation in 23S rRNA (G2576T) leading to linezolid resistance (oxazolidinone class) was also detected. Altogether, Enterococci isolates displayed resistance against nine different antibiotic classes.

### 3.2. Resistance Genes of E. faecalis

**Figure 1:**
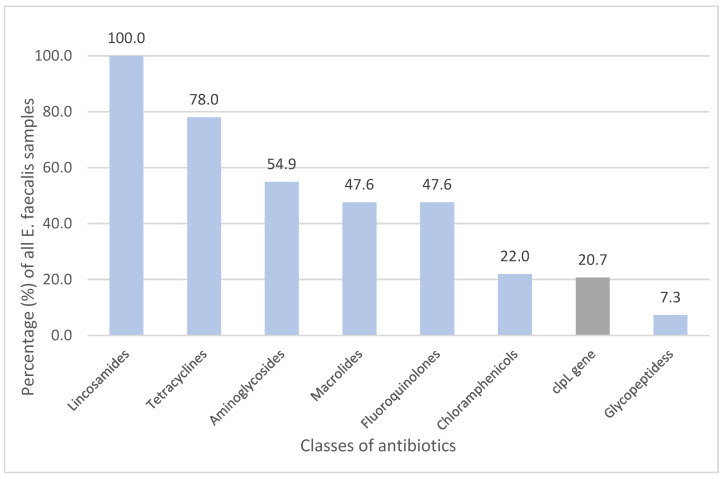
Observed gene resistance against classes of antibiotics in isolates of *E. faecalis* (%).

Isolates of *E. faecalis* carried resistance genes and mutations against seven different classes of antimicrobial agents: lincosamides (100%), tetracyclines (78.0%), aminoglycosides (54.9%), macrolides (47.6%), fluoroquinolones (47.6%), amphenicols (22.0%) and glycopeptides (7.3%) (Figure 1).

The *clp*L gene was observed in 17 out of 82 cases (20.7%). Genetic resistance against lincosamide class drugs, such as clindamycin, was induced by genes *lsa*A, *lsa*E and *lnu*B; furthermore, genes *tet*M and *tet*L were responsible for inducing resistance against tetracyclines.

### 3.3. Resistance Genes of E. faecium

**Figure 2:**
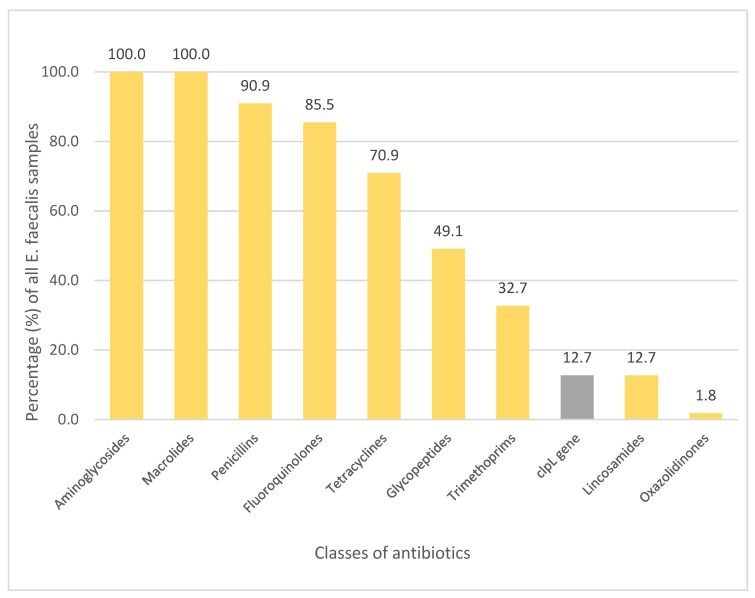
Observed gene resistance against classes of antibiotics in isolates of *E. faecium* (%).

Genes and mutations of *E. faecium* isolates exhibited resistance against nine classes of antimicrobial agents—aminoglycosides (100%), macrolides (100%), penicillin (90.9%), fluoroquinolones (85.5%), tetracyclines (70.9%), glycopeptides (49.1%), trimethoprim (32.7%), lacosamide (12.7%) and oxazolidinones (1.8%). Seven of the fifty-five samples also contained the *clp*L gene (12.7%) (Figure 2).

Aminoglycoside resistance in strains of *E. faecium* was determined by four genes—*aac6′-Ii*, *aac6′-aph2″*, *aph3′-III* and *ant6-Ia*—and macrolide resistance was determined by *msr*C, *erm*B and *erm*T.

### 3.4. Resistance Genes in E. gallinarum, E. durans and E. avium

In samples of *E. gallinarum*, only a resistance against vancomycin (glycopeptide class drug) was detected. Strains of *E. durans* were found to contain the *clp*L gene and have aminoglycoside resistance, whereas *E. avium* was discovered to be multiresistant against aminoglycosides, amphenicols, trimethoprims, macrolides, tetracyclines and glycopeptides.

### 3.5. Incidence of Vancomycin Resistance

**Figure 3:**
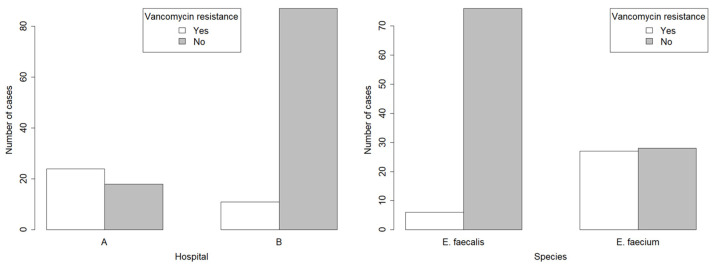
Bar plot A depicts number of positive and negative cases of bacteria tested for vancomycin resistance in samples from two different hospitals (A and B). Hospital A has significantly higher ratio of vancomycin-resistant bacteria (*p*-value < 0.001). Bar plot B depicts number of positive and negative cases of bacteria tested for vancomycin resistance in two *Enterococcus* species (*E. faecalis* and *E. faecium*). *E. faecium* is significantly more vancomycin-resistant than *E. faecalis* (*p*-value < 0.001).

In total, 35 of 140 (25.0%) analysed samples were genetically vancomycin-resistant. Specimens of Enterococci collected at hospital “A” showed vancomycin resistance in 57.1% (n = 24/42) of the cases, whilst in strains isolated from hospital “B” VRE was detected in 11.2% (n = 11/98) of all specimens, which is 5.1 times less in comparison to hospital “A” (*p*-value < 0.001) (plot is on the left of Figure 3). The incidence of VRE between two of the most commonly isolated strains showed significant result differences; 7.3% (6/82) of *E. faecalis* in comparison to 49.1% (27/55) of *E. faecium* samples were vancomycin-resistant (*p*-value < 0.001) (plot is on the right of Figure 3).

*The van*B type was the dominant VRE type, being detected in 25 isolates out of 35 (71.4%). One vancomycin-resistant *van*C type was also detected in the *E. gallinarum* isolate.

Both *van*A and *van*B types contained the regulatory gene *van*RS and resistance genes *van*HAX, *van*HBX and *van*Y, as well as *van*Z (detected only in *van*A types, with the exception of one case found in *E. avium* isolates) and *van*IW (only *van*B types). In 16 *van*B ligase-type resistant samples, resistance genes were located in the Tn1549 transposon.

All vancomycin-resistant samples were multiresistant, containing *gyr*A and *par*C mutations. None of the VRE specimens analysed were detected to carry the *clp*L stress tolerance gene.

### 3.6. Core Genome Analysis

In this stage, only two of the most common species were analysed—*E. faecalis* and *E. faecium.* A relatively large diversity of sequence types (ST) was observed, with *E. faecalis* belonging to 20 different types, the most common being ST6 (n = 20), ST774 (n = 13) and ST179 (n = 10), whilst *E. faecium* belongs to 14 sequence types, with the most common being ST80 (n = 17), ST17 (n = 10) and ST78 (n = 9). Interestingly, almost half (46.2%) of the *E. faecalis* with ST774 had lost their vancomycin resistance genes by the time the isolates were sequenced.

### 3.7. Analysis of E. faecalis

**Figure 4:**
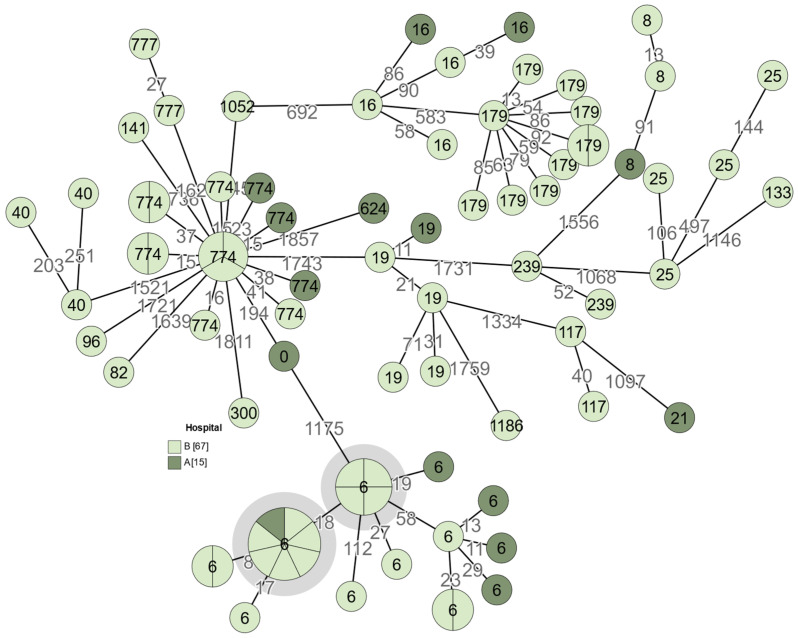
A minimum spanning tree of *E. faecalis* isolates (n = 82). Isolates with 0–7 allelic differences are collapsed into larger nodes. The count of allelic differences between the most similar genotypes is indicated with numbers on the connecting branches. For better visibility, branch lengths are shown in a logarithmic scale. The number on each node represents its sequence type. It was not possible to determine the sequence type of one isolate; therefore, it is indicated as ST0. Grey circles show possible outbreaks of multiresistant but vancomycin-susceptible isolates belonging to ST6. The number of isolates obtained from each hospital is shown in square brackets.

In order to evaluate the genetic relatedness of *E. faecalis* isolates specifically, it is recommended to examine strains with 0–7 allelic differences to call them related [16]. Figure 4 shows that high genetic similarity between four and more isolates can be observed in two clusters (marked in grey), which could indicate a potential outbreak. In one case there are seven related samples and in the other case—four. All of the isolates in these clusters are ST6 and multiresistant; however, vancomycin resistance was not observed in this cluster. This *Enterococcus* outbreak is mainly local to hospital “B” in the year 2022, except for one isolate which was obtained from hospital ”A” in 2021. This might indicate a transmission of pathogenic bacteria between the hospitals.

### 3.8. Analysis of E. faecium

Fifty-one of fifty-five *E. faecium* isolates belonged to clonal complex 17 (CC 17), which represents bacteria acquired in hospitals and have an evolutionary split from commensal bacteria. In order to evaluate closely related *E. faecium* strains, it is advised to focus on cases with 0–20 allelic differences between isolates [17].

**Figure 5:**
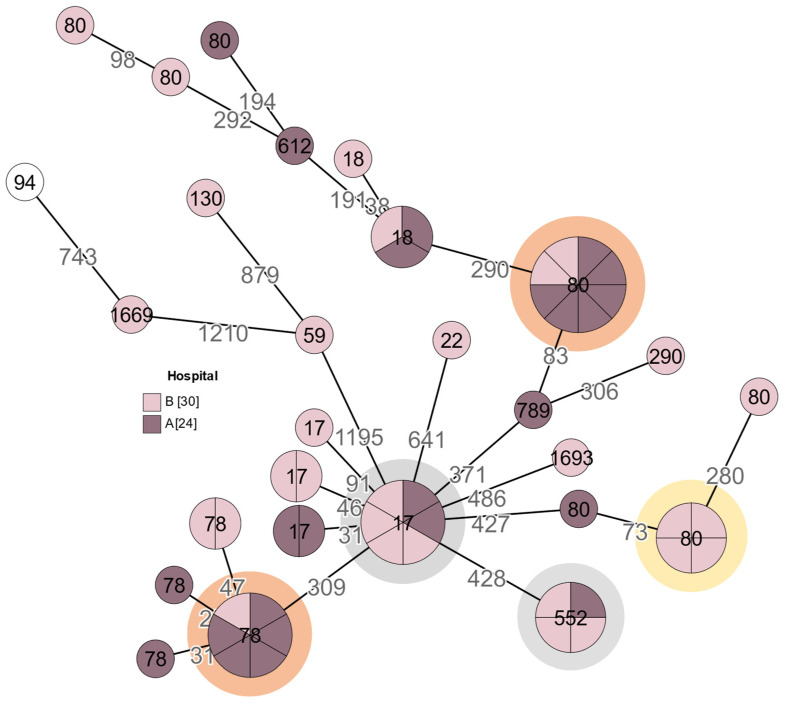
A minimum spanning tree of *E. faecium* isolates (n = 55). Isolates with up to 20 allelic differences are collapsed into larger nodes. The count of allelic differences between the most similar genotypes is indicated with numbers on the connecting branches. For better visibility, branch lengths are shown in a logarithmic scale. The number on each node represents its sequence type. The coloured circles indicate possible outbreaks: Grey—related vancomycin susceptible isolates; Yellow—related *van*A type isolates in hospital “B”; Orange—related *van*B type isolates. The number of isolates obtained from each hospital is shown in square brackets.

In Figure 5, five clusters of related isolates (four and more highly similar isolates) were detected and might indicate an outbreak, with three of the clusters also containing VRE. *Van*B type isolate clusters were observed on two separate occasions, with six isolates belonging to ST78 and eight isolates belonging to ST80, both marked in orange (Figure 5). ST80 isolates in this cluster belonged to the complex type (CT 2579). It is possible to observe that *van*B type resistant Enterococci are mostly dominant within hospital “A” (dark purple spheres). All these samples were collected in 2021.

One possible case of an outbreak with *van*A type resistant Enterococci (marked yellow), was observed in hospital “B”. These specific samples belonged to ST80 (CT2046) and were isolated from urine in 2022. The other two related clusters (grey) were vancomycin susceptible. All of the outbreaks included multiresistant isolates.

## 4. Discussion

Among all isolated *Enterococcus* samples (n = 140), *E. faecalis* (n = 82) and *E. faecium* (n = 55) were the most common species obtained in both hospitals and confirm the statement previously published in the scientific literature [6].

Resistant bacteria can become widespread in the community, if compensatory genetic content is accumulated or resistance gene expression becomes fully inducible upon antibiotic exposure, thus emphasizing the importance of controlling measures to limit the emergence of antimicrobial resistance [18]. The 140 *Enterococcus* isolates collected from the two hospitals revealed that multidrug-resistant Enterococci are a rather significant threat to the hospitals in Latvia. The majority of clinically isolated samples of Enteroccoci were multiresistant, especially isolates of *E. faecium*, which were resistant to at least two antibiotic classes. We discovered that lincosamide class antibiotics are no longer efficient in the treatment of infections caused by *E. faecalis*, and demonstrated that aminoglycoside, macrolide and, in most of the cases, penicillin class antibiotics are ineffective against strains of *E. faecium*.

In our study, we discovered that antimicrobial resistance was induced not only by resistance genes but also by mutations. We observed resistance to three antibiotic classes induced by mutations—fluoroquinolones, penicillins and oxazolidinones. One such mutation was gyrA and parC protein mutations with substitution of one nucleotide in the 83rd and 80th or 87th and 80th (one case) positions in the two mentioned subunit genes. Genes *gyr*A and *par*C encode the production of enzymes—DNA gyrase (gyrA) and topoisomerase IV (parC)—which are essential in the transcription and replication process of DNA and are molecular targets of fluoroquinolone class antibiotics (e.g., ciprofloxacin) [19].

In Gram-positive bacteria, the cell wall is composed of many interconnected layers, which protect bacteria from external hazardous environmental factors. To ensure transglycosylation and transpeptidation, enzymes of the transpeptidase class are required. These are also known as penicillin-binding proteins (PBP). In this study, multiple penicillin-binding protein–5 (PBP-5) mutations were observed in isolates of *E. faecium*. If mutations are found in this protein, it might indicate a higher-level resistance to penicillin drugs, such as ampicillin, due to low adherence, and therefore decreasing the efficacy of ampicillin therapy. Low-level penicillin resistance is innate for most strains of *E. faecium* but is rarely found for strains of *E. faecalis* [20]. Data obtained in this study supports this statement, as these mutations were not observed in any of the analysed samples of *E. faecalis*.

In one case, an isolate had a mutation in the 23S rRNA (G2576T), which promotes the development of resistance against linezolid—a relatively new, synthetic, oxazolidinone class antibiotic. This is the drug of choice for critically ill patients in intensive care units with VRE or methicillin-resistant *Staphylococcus aureus* infections [21]. None of the more prevalent linezolid resistance genes, such as *pox*A, *optr*A or *cfr* were detected.

To verify the accuracy of the data, the antimicrobial susceptibility for this isolate was retested using the broth microdilution method and showed minimal inhibitory concentration (MIC) of 16 µg/mL (R ≥ 4 µg/mL). This sample was *van*B type vancomycin-resistant *E. faecium*, isolated from the material of a central venous catheter at the hospital “A” in 2021. Both of the previously mentioned mutations, which cause resistance against fluoroquinolones and ampicillin, were also observed in this sample; therefore, it was resistant to all nine classes of antimicrobial agents observed in this study. This finding might indicate an increase in resistance against linezolid, further decreasing the possibility of using this antibacterial agent for the treatment of VRE infections. In accordance with the information available to us, this is the first documented case of linezolid resistance in Latvia at the time.

Overall, during the process of genetic analysis, we discovered that a total of 20 anti-microbial resistance genes were detected and in many of the isolates, the *clp*L gene was also detected. It is stated in the scientific literature that isolates harbouring this *clp*L gene have a higher tolerance against stressful conditions, including high temperatures and disinfection, and are more efficient in the production of biofilms [22,23]. This should be taken into consideration when performing cleaning and disinfection of hospital facilities where such bacteria are persisting in such situations; cleaning and disinfection procedures must be performed with careful diligence and cautiousness. During sequence analysis of the *clp*L gene location within the genome, it was mostly detected to be located on mobile genetic elements, which, as mentioned before, eases the transfer of the gene among bacteria and provides an opportunity to “delete” the gene via deletion, if required.

The selection of vancomycin-resistant Enterococci is also creating increasingly high concerns among healthcare professionals. From all 140 samples, 25.0% were vancomycin resistant, which is an alarmingly high rate. In comparison, according to the data published by the European Centre for Disease Prevention and Control in 2022, *E. faecium* isolates with a vancomycin AMR phenotype were observed in 17.2% of all invasive samples tested in EU/EEA (excluding the UK) [24]. Despite the fact that in Europe vancomycin *van*A type resistance has been dominating for a long time, increasing numbers of *van*B type VRE have been seen in the past few years [25,26,27]. In this study, the *van*B type was dominant as well, being found in 25 isolates out of 35 (71.4%). Unlike the *van*A type, which is resistant to vancomycin and teicoplanin, this type is known to be susceptible to the safer antibiotic options between the two—teicoplanin, which causes fewer adverse effects with the same efficacy. In 16 samples with *van*B type resistance (64.0%), the genes were located in the Tn1549 transposon. This supports bacterial adaptability and survival against vancomycin, and also horizontal gene transfer with the *van*B gene between bacteria. One vancomycin-resistant *van*C type was also detected in the *E. gallinarum* isolate. All of the VRE isolates had all of the regulatory genes. Regulatory genes are necessary for bacteria to recognise antibiotics and react accordingly, by initiating responsible genes [28], as without them, resistance would not be possible. This is similar to the *van*HAX/HBX operon—dehydrogenase *van*H, ligase *van*A and *van*B and dipeptidase *van*X—working simultaneously to change the binding site of vancomycin, and therefore prevents it from binding to the cell wall [29].

Enterococci showed a variety of sequence types highlighting the genetic diversity among the species. The dominant sequence type of analysed Enterococci was ST6 (24.4%) and ST80 (30.9%) for *E. faecalis* and *E. faecium,* respectively. Both are known to be hospital-acquired types and one of the predominant clones among CC17 in Europe. Based on the obtained information, it is also possible to conclude that samples of *E. faecalis* with ST6 are more likely to have vancomycin resistance, which also pertains to vancomycin-resistant specimens of *E. faecium* with ST78 and ST80. Interestingly, almost half (46.2%) of the initial VR *E. faecalis* with ST774, had no vancomycin resistance genes at the time of sequencing. This might indicate that this strain of *E. faecalis* is more prone to VR gene deletion after just a few recultivations, unlike other strains.

Regarding *E. faecalis*, two possible outbreaks, mainly in hospital “B”, were observed. Although the strains did not contain vancomycin-resistant bacteria, they still contained multidrug-resistant Enterococci. In the case of *E. faecium,* five related clusters of isolates were detected and might indicate an outbreak. Three of them contain VRE. The *van*B type resistant Enterococci were mostly dominant within hospital “A” and one case of a *van*A type VRE outbreak was noted in hospital “B” in the year 2022.

When comparing EUCAST data for VR *E. faecium* prevalence in Latvia in 2020 (25.0–50.0% of *E. faecium* were VR), the incidence in 2021 and 2022 has remained high; therefore, more strict infection control measures to decrease the emergence of resistant bacteria should be considered and implemented in hospital settings, as well as the number of microbiological susceptibility testing should be intensified for better adjustment of antibacterial therapy. Possibly, one of the reasons for the decreased transmission of VRE might be a more extensive performance of therapeutic drug monitoring procedures; TDM protocol was already used in hospital “B”. In comparison, VRE rates at this hospital were significantly lower than at hospital “A”. However, it must also be noted that the implementation of therapeutic drug monitoring procedures can be challenging, considering the need for a multidisciplinary team, consisting of various healthcare experts, including doctors, clinical pharmacists and microbiologists, to obtain accurate and clinically relevant antibiotic concentrations and ensure appropriate communication among involved healthcare professionals [30], as well as the related economic impact of such approaches [31], to maintain principles of the best clinical practices.

As for the limitations of this study, it is important to understand that although random sampling was used within each hospital, the different sample sizes (42 from A and 98 from B) do not reflect the actual population sizes of these regions. This means our findings apply to the sampled individuals but may not be directly generalizable to the entire hospital’s populations of A and B. We chose this approach to avoid biasing the results towards one population size. However, we recognize that the different sample sizes and lack of proportionality to population size limit the results’ generalizability to broader populations.

## 5. Conclusions

The pilot study of randomly sampled Enterococci samples from patients shows that the incidence of vancomycin-resistant *E. faecium* samples from 2021 to 2022 remains high—49.1% (n = 27/55). The prevalence of vancomycin resistance was highest in the hospital without a therapeutic drug monitoring protocol. The dominating vancomycin resistance phenotype was *van*B. In addition, the *clp*L gene, associated with stress tolerance, including against higher temperatures, and biofilm production, was also frequently observed. A mutation (G2576T) in the 23S rRNA gene was detected, leading to resistance against linezolid which is a currently rare and concerning issue. The most prevalent clinically isolated strains of Enterococci among the two Latvian hospitals are ST6 for *E. faecalis* and ST80 for *E. faecium*, both being multidrug resistant.

## Figures and Tables

**Table 1:** Resistance genes and antibiotic classes identified in all analysed *Enterococcus* isolates (n = 140).

Resistance Gene	Antibiotic Class	Resistance Gene	Antibiotic Class
*aac*(6′)-*aph*(2″)	Aminoglycosides	*cat*(pC221)	Chloramphenicols
*aac*(6′)-*Ii*		*dfr*G	Trimethoprim
*aac*(6′)*-Iih*		*lnu*B	Lincosamides
*ant*(6)-*Ia*		*lsa*A	
*ant*(9)-*Ia*		*lsa*E	
*aph*(3′)-*III*		*tet*L	Tetracyclines
*erm*A	Macrolides	*tet*M	
*erm*B		*van*C1XY	Glycopeptides
*erm*T		*Van*HAX	
*msr*C		*Van*HBX	

## Data Availability

The raw sequence reads from the bacterial isolates included in this study have been deposited in the sequence read archive (SRA) in the National Library of Medicine (NCBI) cloud. BioProject ID: PRJNA1085503.

## Supplementary Materials

The following supporting information can be downloaded at: https://www.mdpi.com/article/10.3390/medicina60060850/s1, Gene analysis and information about samples.
